# Unfavorable perceived neighborhood environment associates with less routine healthcare utilization: Data from the Dallas Heart Study

**DOI:** 10.1371/journal.pone.0230041

**Published:** 2020-03-12

**Authors:** Joniqua N. Ceasar, Colby Ayers, Marcus R. Andrews, Sophie E. Claudel, Kosuke Tamura, Sandeep Das, James de Lemos, Ian J. Neeland, Tiffany M. Powell-Wiley

**Affiliations:** 1 National Heart, Lung and Blood Institute, National Institutes of Health, Bethesda, Maryland, United States of America; 2 University of Texas Southwestern Medical Center, Dallas, Texas, United States of America; Osakidetza Basque Health Service, SPAIN

## Abstract

Neighborhood environment perception (NEP) has been associated with health outcomes. However, little is known about how NEP relates to routine healthcare utilization. This study investigated the relationship between NEP and independent subfactors with healthcare utilization behavior, as measured by self-reported (1) usual source of healthcare and (2) time since last routine healthcare check-up. We used cross-sectional data from the Dallas Heart Study, which features a diverse, probability-based sample of Dallas County residents ages 18 to 65. We used logistic regression modeling to examine the association of self-reported NEP and routine healthcare utilization. NEP was assessed via a questionnaire exploring residents’ neighborhood perceptions, including violence, the physical environment, and social cohesion. Routine healthcare utilization was assessed via self-reported responses regarding usual source of care and time since last routine healthcare check-up. The analytic sample (N = 1706) was 58% black, 27% white, 15% Hispanic, 42% male, and had a mean age of 51 (SD = 10.3). Analysis of NEP by tertile demonstrated that younger age, lower income, and lower education were associated with unfavorable overall NEP (p trend <0.05 for each). After adjustment for potential confounders, including neighborhood deprivation, health insurance, disease burden and psychosocial factors, we found that individuals with more unfavorable perception of their physical environment were more likely to report lack of a usual source of care (p = 0.013). Individuals with more unfavorable perception of the neighborhood physical environment or greater neighborhood violence reported longer time periods since last routine visit (p = 0.001, p = 0.034 respectively). There was no relationship between perceived social cohesion and healthcare utilization. Using a multi-ethnic cohort, we found that NEP significantly associates with report of a usual source of care and time since last routine check-up. Our findings suggest that public health professionals should prioritize improving NEP since it may act as barrier to routine preventive healthcare and ideal health outcomes.

## Introduction

Despite increasing rates of obesity and chronic disease, a significant segment of the United States population does not access routine comprehensive, quality healthcare [1]. According to Healthy People 2020, access to care is defined as “the timely use of personal health services to achieve the best health outcomes” and requires 1)gaining entry into the health care system, 2)accessing a location where needed healthcare services are provided and 3)finding a healthcare provider whom the patient trusts and with whom they can communicate effectively [2]. Access to comprehensive, quality healthcare is important for promoting and maintaining health, preventing and managing chronic disease and reducing unnecessary disability and premature death [2]. For instance, having a usual source of care is a more robust predictor than insurance status for healthcare utilization, cardiovascular disease screening, or receipt of information from healthcare providers about necessary lifestyle changes and inadequate hypertension management [3,4]. For individuals who have experienced an acute myocardial infarction, lack of a usual source of care is associated with higher mortality rates compared to those reporting stronger relationships with a regular physician [5].

**Fig 1:**
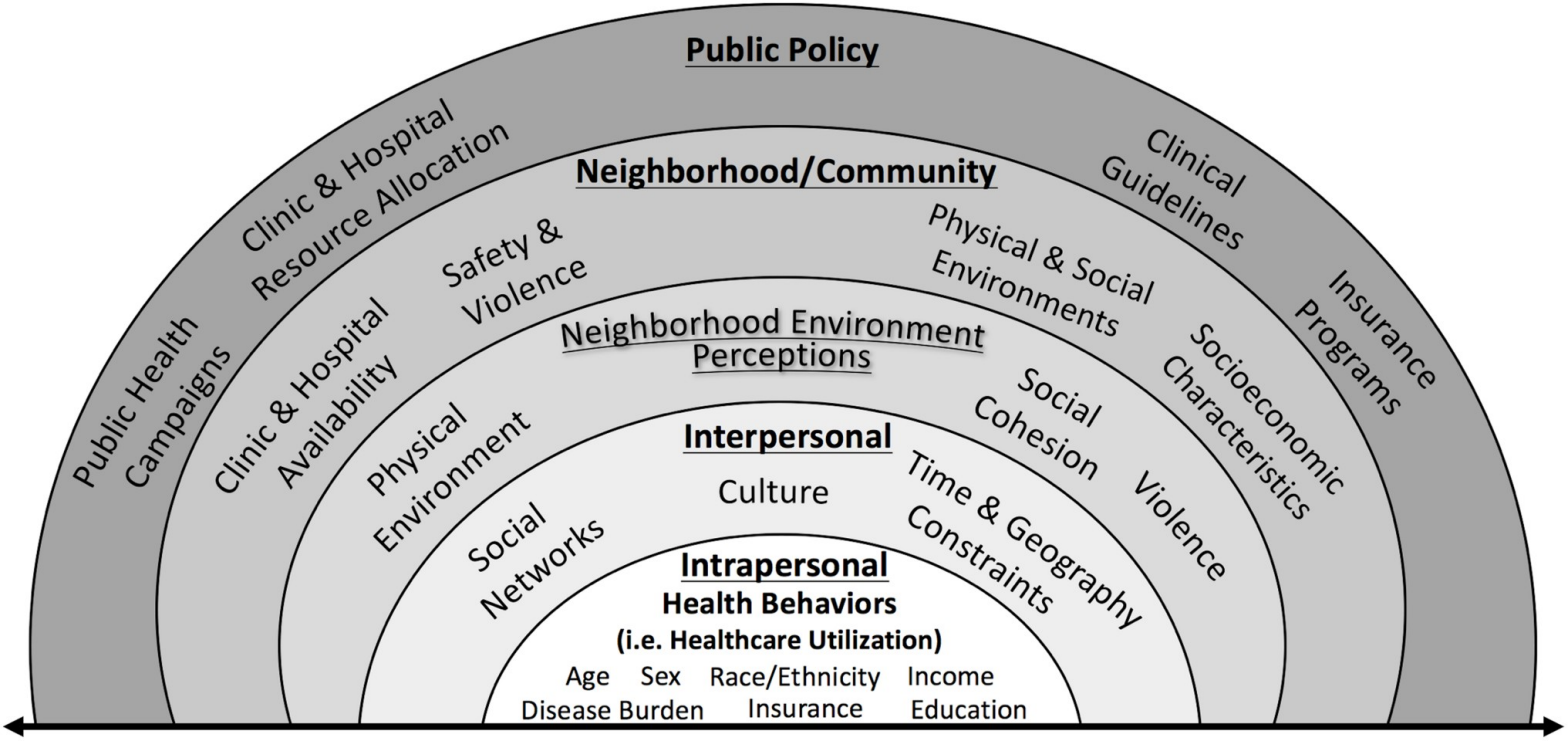
The socio-ecological model for health behavior. This modified socio-ecological model introduces the influence of both objective and perceived neighborhood factors which may be associated with an individual’s health behaviors, including healthcare utilization.

Socio-ecological models of health outcomes emphasize how factors at the intrapersonal, interpersonal, and community level greatly affect health behaviors and care access [6]. For example, healthcare utilization disparities are not only related to individual race/ethnicity, but are also significantly related to the racial/ethnic composition of one’s neighborhood [7,8]. Furthermore, it has been well-documented that an individual’s perceptions regarding neighborhood environment associates with cardiovascular risk factors including physical activity [9–12]. Additionally, cross-sectional data has shown that concern for neighborhood problems and disorder is correlated with higher blood pressure, higher BMI, and increased likelihood of tobacco use, all of which negatively associate with ideal cardiovascular health [13,14]. In contrast, more favorable perceptions of social cohesion have been associated with decreased likelihood of tobacco use [15,16]. These associations inform the development of a socio-ecological model specifically tailored to healthcare utilization behavior (Fig 1). We modified McLeroy’s socio-ecological model to explicitly highlight the role of one’s objective and perceived neighborhood factors, since both may be associated with health behavior [17]. Perceptions of neighborhood violence, the physical environment, and social cohesion may act as facilitators or barriers in the decision to participate in routine healthcare and may be distinct from objective measures of an individual’s surroundings. An individual’s intra- and interpersonal factors interplay with the neighborhood environment to influence his or her perceptions which may contribute to lifestyle and health.

Limited research has explored the relationship between neighborhood environment perception (NEP) and healthcare utilization [18–20]. Aysola et al found that children of parents with more unfavorable NEP regarding violence, physical environment and social cohesion were less likely to receive primary care from a patient-centered medical home [18]. For adults, limited data demonstrate an association between social cohesion and healthcare utilization behavior, but the data are not generalizable given the homogeneity of a majority white cohort [19,20]. Further investigation is necessary to determine whether NEP, despite objective neighborhood quality, plays a significant influence in healthcare utilization, especially within a diverse cohort. This information may be helpful for healthcare providers and policy makers hoping to improve health among historically underserved communities who disproportionately live in low-income and limited resource environments with greater neighborhood disorder.

With a socio-ecological framework (Fig 1), we examined the relationship between NEP and self-reported healthcare resource utilization for an urban, community-based cohort in Dallas County, Texas using data from the Dallas Heart Study (DHS). It was expected that individuals with more unfavorable NEP (total and subfactors) would report lower healthcare utilization rates.

## Methods

### Study population

The DHS cohort is a single-site, diverse, probability-based sample of Dallas County residents aged 18 to 65 at study entry with data collection from 2000–2002. Since the question of interest was only asked in the survey administered to follow-up participants from 2007–2009, we were limited to that dataset. Non-Hispanic blacks were intentionally over-sampled to compose half of the study population, and detailed data collection methods were reported previously [21]. At study entry, 3072 participants completed a detailed survey and phenotypic (i.e. height, weight, biomarkers) measurements. A total of 2485 of these participants underwent follow-up testing during a single visit to the University of Texas (UT) Southwestern Medical Center between September 2007 and December 2009. The DHS protocol was approved by the UT Southwestern Medical Center’s Institutional Review Board; all study participants provided written informed consent at study entry and follow-up. A protocol for DHS neighborhood environment data analyses (13-H-N041) was approved by the Institutional Review Board of the National Heart, Lung, and Blood Institute of the National Institutes of Health.

**Fig 2:**
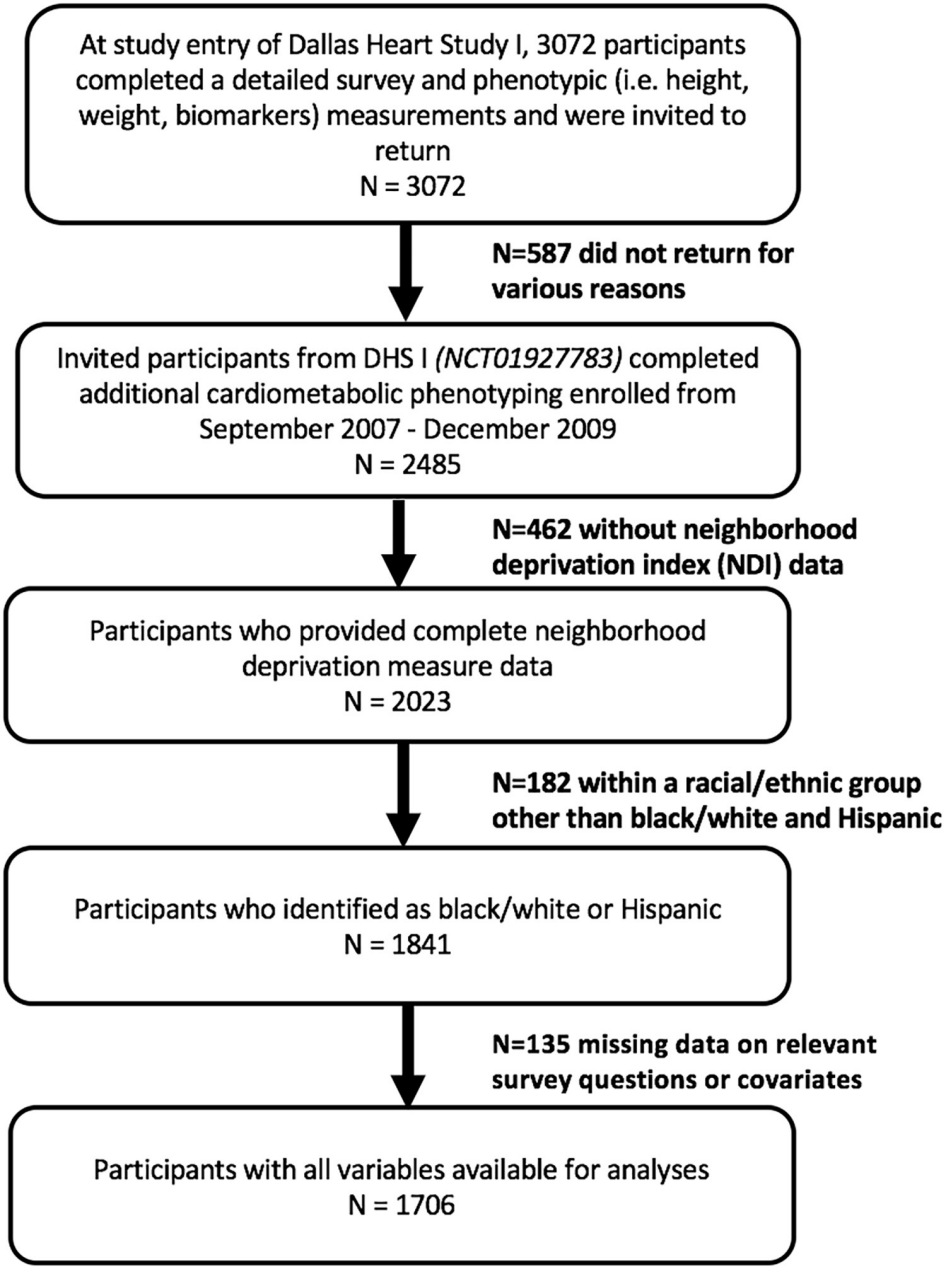
CONSORT diagram describing analytic sample.

For this study, we used data from individuals who participated in follow up testing (N = 2485). We excluded those who were missing neighborhood deprivation measure data (N = 462), those of a racial/ethnic group other than black/white and Hispanic (N = 182) and those missing data on relevant survey questions or covariates (N = 135). Exclusions resulted in a final analytic sample of 1706 (Fig 2). As a standard practice for publications from the Dallas Heart Study, participants whose race/ethnicity was other than black/white or Hispanic were excluded from analyses [22–24]. Individuals that fall into the “other” category compose a small heterogeneous sample of the study population. The sample size for this part of the cohort limits the statistical power available for drawing group-specific conclusions about study findings. A comparison of the excluded and analytic samples can be found in S3 Table.

### Exposure: Neighborhood environment perception (NEP)

The questions used to assess individuals’ NEP were derived from the 1994 Project on Human Development in Chicago Neighborhoods [25]. Participants answered 18 Likert-scale questions regarding neighborhood environment perceptions. The responses were standardized on a scale of one to five with a higher score representing a more unfavorable perception of that specific characteristic.

Principal components factor analysis with varimax (orthogonal) rotation was used to define constructs based on 18 questions, as previously described [26]. Three separate factors were identified within the construct of NEP: 1)perceived neighborhood violence, 2)perceived physical environment, and 3)perceived social cohesion. Perceived violence was described via reports of frequency of crime within the past six months and feelings of safety via response options of “never”, “rarely”, “sometimes” and “often”. Physical environment perception characterized individuals’ thoughts on neighborhood physical characteristics including seriousness of noise, traffic, lack of access to food shopping, lack of recreational opportunities, and trash and litter using a scale of one to ten; a response of one indicated it “not really a problem” while ten indicated “a very serious problem”. Social cohesion captured feelings that one resided within a close-knit neighborhood with neighbors who are willing to help others, can be trusted, and share the same values. Participants could answer whether they “strongly agree”, “agree”, disagree” or “strongly disagree” with statements evaluating social cohesion.

Principal components factor analysis with varimax (orthogonal) rotation was used to define constructs or factors from the neighborhood questionnaire data. Neighborhood questions with a loading score of 0.40 or higher were used to define the theme of each factor, since meeting this criteria strongly suggests that a specific variable can be attributed to a factor [27]. The eigenvalue was used as a measure of variance explained by variables comprising each factor and had to be greater than or equal to one. Cronbach’s alpha coefficients were used to measure internal consistency of the variables in each factor, with values above 0.7 considered acceptable. Numeric values assigned to Likert scale answers for a factor’s questions were summed to calculate a factor-related perception score; a total NEP was the sum of factor-related perception scores.

### Routine healthcare utilization outcomes

Routine healthcare utilization was based on questions derived from the 1999 Behavioral Risk Factor Surveillance System [28]. This study analyzed two main questions regarding healthcare behavior: 1)“Is there one particular clinic, health center, doctor’s office or other place that you usually go to when you need a routine exam, are sick, or need advice about your health?” and 2)“About how long has it been since you last visited a clinic, health center, doctor’s office, or other medical facility for a routine check-up?”

The question investigating usual source of care allowed for five possible responses, including the response “yes, one particular place” (referent group) to which the responses “yes, but more than one place” and “no” were compared. We classified these responses into a three-level categorical outcome by eliminating two of the answers in the analysis: “don’t know” and “refused”. Those who answered “yes” were invited to answer a follow up question, “What kind of place is it? Would you say it is….” that presented eight possible answer choices including a doctor’s office, hospital, or some other kind of place.

The question exploring duration of time since last routine check-up allowed for seven possible responses. The response “within the past year” was the referent group to which the responses “within the past two years”, “within the past five years”, and “five or more years ago” and “never” were compared. We classified these responses into a four-level categorical outcome by eliminating responses “don’t know/not sure” and “refused” from analyses and combining “never” and “five or more years ago” into one category.

### Covariates

Demographics included: self-reported age in years, sex, race/ethnicity, household income, education, and marital status. In addition, survey data provided self-reported health insurance status, depression and experience of racism. Depression was measured via the Quick Inventory of Depressive Symptomatology (QIDS) [29]. Experiences of racism was measured by two survey questions which asked if the participant had ever been discriminated against because of race/ethnicity in general or when receiving medical care. Presence of cardiovascular disease was determined by diagnosis of hypertension, diabetes, or self-reported history of cardiovascular disease. Hypertension was defined as one of the following: systolic blood pressure ≥ 140 mmHg, diastolic blood pressure ≥ 90 mmHg, or the use of anti-hypertensive medication. Diabetes mellitus was defined by hemoglobin A1c value measured in DHS II, and included those with a hemoglobin A1c ≥ 6.5%. Diabetes mellitus was also defined by either by self-report accompanied by use of anti-hyperglycemic medication or by fasting serum glucose ≥ 126 mg/dl for participants with a hemoglobin A1c value below the cut-off. Self-reported comorbid disease burden was defined as “yes” if a participant reported one or more diagnoses of emphysema, cancer, and rheumatoid arthritis. Finally, a block group-level neighborhood deprivation index (NDI) was developed for Dallas County using variables from the 2000 U.S. Census, as described previously [30,31]. Neighborhood variables incorporated into the NDI were in six domains (education, employment/occupation, housing conditions, income/poverty, racial composition, and residential stability). Six variables were selected to compute the NDI score (% unemployment, % female-headed households, % households on public assistance, % households with a car, % the population below the federal poverty line, and % non-Hispanic blacks) after being standardized and weighted by their factor loading coefficients. NDI score was analyzed as a continuous variable and categorized into tertiles of low, medium, or high neighborhood-level socioeconomic deprivation. Some covariates could be characterized as potential moderators (i.e. sex, race/ethnicity), prompting assessment of sex and race interactions.

### Statistical analysis

Baseline characteristics were compared across tertiles of NEP using Jonkheere-Terpstra analysis of trend as described previously [26,31–34]. Sensitivity analyses were done to account for the analytic sample size due to exclusions of participants who were missing covariates and/or answered “don’t know” or “refuse” to survey items of interest. Sensitivity analyses included use of a Markov Chain Monte Carlo algorithm for imputing the missing data. This form of Rubin’s multiple imputation procedure results in each missing value being replaced with a set of plausible values that represent the uncertainty in the value. We created 5 imputed datasets and the standard errors were adjusted after combining the datasets to reflect the within-imputation variance.

Principal components factor analysis with varimax (orthogonal) rotation was used to define constructs or factors from the neighborhood questionnaire data as described previously [26].

Adjusted logistic regression models were employed to examine associations between NEP and NEP subfactors with usual source of care. Individuals who reported one particular source of care comprised the referent group. Adjusted logistic regression models were used to assess odds ratios of routine health check-ups with individuals who reported a routine check-up within the past year being the referent group. Fully adjusted models accounted for age, sex, race/ethnicity, income, education, NDI, marital status, health insurance status, disease burden, depression and experience of racism as covariates. Additionally, to confirm that NEP was the driving force, we estimated models with NEP only, NDI only, and NEP and NDI together to assess how the variables relate to healthcare utilization individually and jointly. Finally, interaction terms were analyzed with regards to race/ethnicity and an analysis of covariates was done to ensure proper adjustment and no multicollinearity. All of the variance inflation factors used in the analyses were less than 4 to evaluate for any multicollinearity [35].

Analyses were performed using Statistical Analysis Software (version 9.4, SAS Institute Inc., Cary, NC) with an a priori alpha level of 0.05.

## Results

With regards to ensuring the analytic sample was appropriate for drawing conclusions, imputation results suggested that there was little impact on study results with regards to total NEP, as shown in S1 and S2 Tables. Additionally, all of the variance inflation factors are <4 indicating that multicollinearity is not an issue for the analyzed covariates in the models.

Table 1 shows baseline characteristics for the study population across tertiles of total NEP. Total NEP score ranged from 10–47. The study population consisted of 58% non-Hispanic blacks, 27% non-Hispanic whites, and 15% Hispanics. Individuals with the most unfavorable NEP were more likely to be black, have lower education and income levels, and live in areas with higher NDI (p trend ≤0.04 for all). Furthermore, this group was also found to have higher prevalence of cardiovascular risk factors including a history of hypertension (p <0.05).

**Table 1:** Demographics, socioeconomic status, neighborhood deprivation index, insurance status, marital status, and cardiovascular risk factors across tertiles of total neighborhood environment perception (NEP) score for participants in the Dallas Heart Study (N = 1706) (2007–2009). Tertile 3 with most unfavorable NEP.

Characteristic	Tertile 1 N = 518	Tertile 2 N = 578	Tertile 3 N = 610	p trend
Score range	10–14	15–19	20–47	
**General Demographics**				
Age (years), mean (SD)	52.06 (10.63)	50.93 (10.10)	50.70 (10.24)	0.04
Male, N (%)	221 (42.7)	268 (46.4)	22 (37.1)	0.04
Black, N (%)	270 (52.1)	313 (54.2)	395 (65.8)	<0.0001
White, N (%)	153 (29.5)	178 (30.8)	133 (21.8)	0.003
Hispanic, N (%)	95 (18.3)	87 (15.1)	82 (13.4)	0.03
**Marital Status**				
Yes, N (%)	284 (55.3)	280 (49.0)	208 (34.8)	<0.0001
**Socioeconomic Status**				
***Education***				
Less than High School, N (%)	76 (14.7)	87 (15.1)	123 (20.2)	0.01
High School, N (%)	116 (22.4)	168 (29.2)	192 (31.6)	0.0007
Some College, N (%)	272 (52.5)	277 (48.1)	262 (43.8)	0.003
College or higher, N (%)	54 (10.4)	44 (7.6)	27 (4.4)	0.0001
***Income***				
<$16,000, N (%)	69 (14.5)	94 (17.7)	165 (29.8)	<0.0001
$16,000 − $29,999, N (%)	78 (16.4)	104 (19.6)	116 (21.0)	0.07
$30,000 − $49,999, N (%)	132 (27.8)	153 (28.9)	150 (27.1)	0.79
>$50,000, N (%)	196 (41.3)	179 (33.8)	122 (22.1)	<0.0001
**Neighborhood Deprivation Index**				
Low, N (%)	217 (41.9)	217 (37.5)	119 (19.5)	<0.0001
Intermediate, N (%)	179 (34.6)	205 (35.5)	190 (31.2)	0.21
High, N (%)	122 (23.6)	156 (27.0)	301 (49.3)	<0.0001
**Insurance Status**				
Yes, N (%)	387 (75.0)	434 (75.7)	430 (71.1)	0.12
**Cardiovascular Risk Factors**				
History of hypertension, N (%)	267 (51.5)	306 (52.9)	351 (57.5)	0.04
History of diabetes, N (%)	82 (15.8)	100 (17.3)	117 (19.2)	0.14
History of CVD, N (%)	26 (5.0)	34 (5.9)	46 (7.5)	0.08

SD = Standard Deviation, CVD = Cardiovascular Disease

Table 2 shows routine healthcare utilization as measured by 1)usual source of care (quantity and type) and 2)time since last routine healthcare check-up across tertiles of total NEP. Individuals with the most unfavorable NEP were more likely to report having no usual source of care and less likely to report one particular source of routine care (p <0.05). Furthermore, those with more unfavorable NEP who did report a usual source of care were less likely describe their source as a doctor’s office (p <0.001). Individuals with more unfavorable NEP were also more likely to report that their last routine check-up had been within the past five years (p = 0.01) or greater than five years/never (p = 0.04).

**Table 2:** Report of usual source of care (quantity and type) and routine healthcare utilization across tertiles of total neighborhood perception score for participants in the Dallas Heart Study (N = 1706) (2007–2009).

	Tertile 1 N = 518	Tertile 2 N = 578	Tertile 3 N = 610	p trend
Score range	10–14	15–19	20–47	
**Usual source of care: quantity**				
None, N (%)	74 (14.4)	85 (14.7)	115 (19.0)	0.03
One particular place, N (%)	422 (81.9)	470 (81.5)	461 (76.3)	0.02
More than one place, N (%)	19 (3.7)	22 (3.8)	28 (4.6)	0.41
**Usual source of care: type**				
Doctor’s office, N (%)	323 (73.4)	342 (70.2)	302 (62.3)	0.0002
Clinic or health center, N (%)	98 (22.3)	114 (23.4)	124 (25.6)	0.24
Hospital or outpatient department, N (%)	14 (3.2)	26 (5.3)	48 (9.9)	<0.0001
Hospital emergency room, N (%)	3 (0.7)	2 (0.4)	3 (0.6)	0.92
Urgent care center, N (%)	2 (0.5)	2 (0.4)	4 (0.8)	0.44
Some other kind of place, N (%)	0 (0.0)	1 (0.2)	4 (0.8)	0.03
**Time since last routine check-up**				
Within the past year, N (%)	392 (76.3)	408 (71.1)	417 (69.3)	0.01
Within the past two years, N (%)	64 (12.5)	75 (13.1)	73 (12.1)	0.85
Within the past 5 years, N (%)	21 (4.1)	34 (5.9)	46 (7.6)	0.01
5 or more years ago or never, N (%)	37 (7.2)	57 (9.9)	66 (11.0)	0.04

As shown in Table 3 for every one unit increase in the NEP point score (one unit increase corresponds with one point increase), the odds of reporting no usual source of care were 1.18 times higher than reporting one particular source of care (95% Confidence Interval (CI) 1.00–1.40), and was marginally significant. In the fully adjusted model, those who reported a more unfavorable perceived physical environment, had 1.24 (95% CI 1.05–1.46) higher odds of reporting no usual source of care compared to those reporting one specific source.

**Table 3:** Odds ratios of reporting a usual source of care as related to neighborhood environment perception. Reference group reports having one usual source of care. Final model adjusted for age, sex, race/ethnicity, marital status, income, education, neighborhood deprivation index, insurance status, cardiovascular disease, comorbid disease burden, depression and experience of discrimination.

	Odds Ratio Estimate	Confidence Interval

**Total Neighborhood Environment Perception**		
Yes, one place	Reference Group	
Yes, more than one place	1.07	0.79–1.44
None	**1.18**	**1.00–1.40**

**Factor 1: Perceived Violence**		
Yes, one place	Reference Group	
Yes, more than one place	1.04	0.88–1.22
None	0.99	0.73–1.34

**Factor 2: Perceived Physical Environment**		
Yes, one place	Reference Group	
Yes, more than one place	1.20	0.91–1.58
None	**1.24**	**1.05–1.46**

**Factor 3: Perceived Social Cohesion**		
Yes, one place	Reference Group	
Yes, more than one place	0.90	0.68–1.21
None	1.05	0.89–1.25

As shown in Table 4, for every one unit increase in NEP, the odds of reporting a last routine check-up within the past two to five years were 1.33 (95% CI 1.05–1.67) times higher when compared to reporting a check-up within the past year. Similarly, for every unit increase in NEP, the odds of reporting a routine visit that had occurred five or more years ago/never was 1.42 (95% CI 1.16–1.75) times higher than reporting a routine visit in the past year. Table 4 further shows the relationship between routine check-up and NEP subfactors (neighborhood violence, physical environment and social cohesion). Individuals with more unfavorable perceptions about neighborhood violence were 1.32 (95% CI 1.07–1.64) times more likely to report their last routine check-up as being within the past two to five years in comparison to the referent group. In addition, individuals with more unfavorable perceptions of their neighborhood physical environments had a 1.29 (95% CI 1.03–1.61) times higher odds of reporting that their last routine check-up was within the past two to five years as compared to within the past year.

**Table 4:** Odds ratios of reporting routine check-up as related to neighborhood environment perception and subfactors. Reference group reports most recent routine check-up within past year (0–12 months). Final model adjusted for age, sex, race/ethnicity, marital status, income, education, neighborhood deprivation index, insurance status, cardiovascular disease, comorbid disease burden, depression and experience of discrimination.

	Odds Ratio Estimate	Confidence Interval

**Total Neighborhood Environment Perception**		
0–12 months	Reference Group	
1–2 years	1.05	0.87–1.27
2–5 years	**1.33**	**1.05–1.67**
More than 5 years or Never	**1.42**	**1.16–1.75**

**Factor 1: Perceived Violence**		
0–12 months	Reference Group	
1–2 years	1.14	0.96–1.36
2–5 years	**1.32**	**1.07–1.64**
More than 5 years or Never	1.20	0.98–1.47

**Factor 2: Perceived Physical Environment**		
0–12 months	Reference Group	
1–2 years	1.02	0.85–1.22
2–5 years	**1.29**	**1.03–1.61**
More than 5 years or Never	**1.44**	**1.19–1.75**

**Factor 3: Perceived Social Cohesion**		
0–12 months	Reference Group	
1–2 years	0.96	0.81–1.14
2–5 years	1.10	0.87–1.40
More than 5 years or Never	1.19	0.96–1.48

Interactions regarding race/ethnicity were not statistically significant (p>0.05). We have also included S3 Table to demonstrate the differences between the excluded sample and analytic sample. The excluded sample has lower NEP scores, higher socioeconomic status, and lower rates of hypertension and diabetes (p<0.05 for all). In addition, the excluded group has a smaller percentage of Black participants, larger percentage of White participants, and smaller percentage of Hispanic participants (p<0.01 for all). Between the two groups there are no significant differences in age or marital status.

With regards to strength of the relationship being dependent on NEP or NDI, S4–S7 Tables illustrate that NEP is driving the relationship since models without this factor are insignificant. The authors have also included the results of the principal components factor analysis in S8 Table.

## Discussion

Results from the cross-sectional analysis of a large, diverse, population-based sample from Dallas County, Texas highlight the complex relationship between perception of neighborhood characteristics and resident healthcare utilization. Residents who report more unfavorable NEP were found to have decreased rates of routine healthcare utilization, as measured by reported engagement with a usual source of care and time since last routine healthcare check-up. Our study is one of the first to investigate how NEP is related to healthcare engagement and utilization.

Our findings contribute to the literature in four main ways. First, our finding that NEP has a significant association with healthcare utilization, even when considering neighborhood-level socioeconomic deprivation, is important for understanding how place influences health. Research has shown low concordance between the perceived and objective built environment, suggesting that these measures may be related but have unique influences on health behavior and should be investigated separately [10,36,37]. Our study is one of the first to demonstrate a relationship between the perceived environment and routine healthcare check-ups. This suggests that efforts to increase healthcare utilization (e.g. increase availability of healthcare resources) without addressing NEP may be limited in their impact on healthcare engagement and utilization.

Second, individuals with more unfavorable perception of their physical environment (e.g. higher perceived presence of noise, traffic, and litter coupled with few parks or playgrounds) are more likely to lack a usual source of care and have longer periods of time between routine healthcare visits. Engagement with a healthcare site for routine check-ups may be due to limited nearby locations as supported by the finding that individuals with highest NEP scores were less likely to report a doctor’s office as their source of care. Primary care physicians are less likely to provide services in low-income and predominately minority communities, further exacerbating obstacles for historically marginalized populations and their access to care [8,38]. Physicians are less likely to establish clinics in low-income neighborhoods as they may be perceived as undesirable and less profitable in comparison to higher income neighborhoods [39]. Individuals residing in neighborhoods with higher perceived disorder may also have a more negative lens regarding availability and accessibility of these opportunities, regardless of objective accessibility. Literature also suggests that having a usual source of care in conjunction with a trusted usual provider may be more influential in obtaining preventive care or screening services when compared to having only a place or neither [40]. It is important that individuals at risk of disproportionately poor health outcomes have a usual source of care, but our findings show that an unfavorable neighborhood perception of the physical environment may serve as a hindrance in establishing and annually accessing medical care.

Third, the significant relationship between perceived violence and time since last routine check-up suggests that individuals could have safety concerns when deciding to attend health maintenance visits. Although no prior literature has explored this particular relationship, there are data indicating that perceptions of neighborhood safety and crime are significantly associated with lower utilization of health-enabling resources, such as large grocery stores, fitness centers and pharmacies [41]. Additionally, Tung and colleagues found that individuals living in high violent crime areas expressed difficulty in balancing challenges imposed by community violence with the demands of living with and managing their chronic conditions [42]. Another study highlighted that adults with type 2 diabetes who reported living in unsafe neighborhoods were more likely to delay prescription refills [43]. Individuals with more safety concerns may have less of an impetus, time or resources to focus on nonemergent care. While a resident may overcome these concerns in the context of acute or emergent healthcare needs, there may be less incentive to confront these barriers for routine healthcare check-ups. Higher perceived violence may also contribute to a lack of healthcare resources as businesses and healthcare professionals are deterred from establishing practices in the area [39]. Travel to and from clinic visits may also serve an obstacle due to concerns of being harmed during transport or general fears of navigating the neighborhood, but this rationale should be further explored.

Finally, this study is one of the first to report no significant relationship between perceived social cohesion and healthcare utilization patterns for a multi-racial cohort. Prior analyses featuring majority white cohorts have revealed that higher perceived social cohesion is positively associated with increased frequency of dental visits and receipt of health screening services [19,20]. However, current literature examining diverse populations reveals inconsistent relationships between social cohesion and health behaviors such as tobacco use, alcohol consumption, medication management and physical activity [15,16,44–47]. The influence of social cohesion may vary according to composition of the study sample and its measurement may vary according to survey instrument tools. Due to cultural diversity, instruments measuring social cohesion may not be as reliable for populations that are racially, ethnically and socioeconomically diverse.

It is noteworthy that the excluded population (eliminated due to missing covariates) and the analytic sample have significant differences. As shown in S3 Table, the excluded population had a better health risk profile with lower rates of hypertension and diabetes. In addition, the excluded population had significant higher income, education and insurance rates. The elimination of this group with more socioeconomic advantages may limit our insight into the relationship between neighborhood perception and healthcare utilization. Previous literature has elucidated that there is a significant relationship between individual level socioeconomic status, neighborhood perception and self-rated health, suggesting an interplay between these factors [48]. For this reason, it is important to analyze a population representing a wide spectrum in socioeconomic levels to better understand how neighborhood perception may relate to health behaviors. However, we are encouraged that despite having to exclude a portion of the sample due to incomplete data, our sensitivity analyses suggest a minimal impact on our study results.

### Strengths and limitations

Noteworthy strengths of this study include the utilization of a multi-racial, urban cohort and being the first of its kind to evaluate the relationship of NEP with healthcare utilization. The primary limitation is that the study excludes a significant portion of the population due to missing variables of interest, which impacts the sample size. The study also excludes those who are not Black, White, or Hispanic in the Dallas Heart Study. More studies should be done with a larger and more heterogeneous and racially/ethnically diverse population to further explore these relationships of interest. The cross-sectional design limits the ability to assess for causality, while reliance on self-reported data risks inaccurate recall and social desirability bias. Furthermore, the lack of objective measurement of available health resources (i.e., hospitals, clinics) and actual health behaviors (i.e., visits per year) limits comparison of the influence of perceived versus objective resources on actual utilization. Finally, questions soliciting the frequency of specific screening activities and preventive health behaviors such as influenza vaccinations, pap smears, mammograms, colonoscopies, and prostate exams were not asked.

## Conclusion

This study contributes to a growing body of literature demonstrating how neighborhood factors may influence health through healthcare utilization. Healthcare professionals working to promote health in limited resource neighborhoods must acknowledge social factors influencing health and health behavior. As many professional organizations express concern for an inevitable primary care physician shortage [49] it becomes especially important that public health officials work to increase the number and frequency of routine healthcare visits for communities with disproportionate disease burden and barriers to healthcare. Our findings suggest that policymakers should seek ways to not only improve the neighborhood conditions and resources, but also work to improve resident perceptions which could increase healthcare utilization rates. More research is recommended to further explore the exact mechanism by which unfavorable NEP impacts routine healthcare utilization for improved health outcomes. These data may equip researchers and policymakers working to enhance health promotion programs targeting communities with high neighborhood disorder. It may be important for public health professionals to target unfavorable neighborhood perception and find ways to improve neighborhoods by not only objective measures, but by subjective components as well, to eliminate barriers to routine preventive healthcare.

## Supporting information

S1 Table Imputation analyses for usual source of care. (DOCX)

S2 Table Imputation analyses for time since last routine visit. (DOCX)

S3 Table Differences between included and excluded participants. (DOCX)

S4 Table Odds ratios of reporting a usual source of care as related to neighborhood deprivation index. Reference group reports having one usual source of care. Model adjusted for age, sex, race/ethnicity, marital status, income, education, neighborhood deprivation index, insurance status, cardiovascular disease, comorbid disease burden, depression and experience of discrimination.
(DOCX)

S5 Table Odds ratios of reporting a usual source of care as related to neighborhood environment perception. Referent group reports having one usual source of care. Model adjusted for age, sex, race/ethnicity, marital status, income, education, insurance status, cardiovascular disease, comorbid disease burden, depression and experience of discrimination.
(DOCX)

S6 Table Odds ratios of reporting routine check-up as related to neighborhood deprivation index. Reference group reports most recent routine health check-up within past year (0–12 months). Model adjusted for age, sex, race/ethnicity, marital status, income, education, neighborhood deprivation index, insurance status, cardiovascular disease, comorbid disease burden, depression and experience of discrimination.
(DOCX)

S7 Table Odds ratios of reporting routine check-up as related to neighborhood environment perception and subfactors. Reference group reports most recent routine health check-up within past year (0–12 months). Model adjusted for age, sex, race/ethnicity, marital status, income, education, insurance status, cardiovascular disease, comorbid disease burden, depression and experience of discrimination.
(DOCX)

S8 Table Factor analysis for creation of neighborhood-related factors in the Dallas Heart Study. (DOCX)
