# Supplementary material for: Unfavorable perceived neighborhood environment associates with less routine healthcare utilization: Data from the Dallas Heart Study
Source: PLoS One. 2020 Mar 12;15(3):e0230041. doi: 10.1371/journal.pone.0230041 (PMC7067436; doi:10.1371/journal.pone.0230041)
Supplement: S1 Table — (DOCX) [file pone.0230041.s001.docx]

**Supplemental Table 1. Imputation Analyses for Usual Source of Care**

|  | Odds Ratio Estimate | Confidence Interval | P value |
| --- | --- | --- | --- |
|  |  | | |
| Violence | 0.93 | 0.78 – 1.11 | 0.39 |
| Physical Environment | 1.2 | 1.03 – 1.47 | 0.02 |
| Social Cohesion | 0.97 | 0.81 – 1.15 | 0.70 |
