# Supplementary material for: Unfavorable perceived neighborhood environment associates with less routine healthcare utilization: Data from the Dallas Heart Study
Source: PLoS One. 2020 Mar 12;15(3):e0230041. doi: 10.1371/journal.pone.0230041 (PMC7067436; doi:10.1371/journal.pone.0230041)
Supplement: S2 Table — (DOCX) [file pone.0230041.s002.docx]

**Supplemental Table 2. Imputation Analyses for Time Since Last Routine Visit**

|  | Odds Ratio Estimate | Confidence Interval | P value |
| --- | --- | --- | --- |
|  |  | | |
| Violence | 1.39 | 1.11 – 1.72 | 0.003 |
| Physical Environment | 1.34 | 1.05 – 1.70 | 0.02 |
| Social Cohesion | 1.06 | 0.87 – 1.29 | 0.55 |
