# Supplementary material for: Unfavorable perceived neighborhood environment associates with less routine healthcare utilization: Data from the Dallas Heart Study
Source: PLoS One. 2020 Mar 12;15(3):e0230041. doi: 10.1371/journal.pone.0230041 (PMC7067436; doi:10.1371/journal.pone.0230041)
Supplement: S3 Table — (DOCX) [file pone.0230041.s003.docx]

**Supplemental Table 3. Differences between Included and Excluded Participants**

| **Characteristic** | **Included (n=1706)** | **Excluded (n=779)** | **P trend** |
| --- | --- | --- | --- |
| **NEP mean, SD** | 18.92 (6.77) | 17.68 (6.13) | <0.0001 |
| **Age mean, SD** | 51.19 (10.32) | 50.81 (10.33) | 0.56 |
| **NDI mean, SD** | 0.48 (1.2) | 0.37 (1.17) | 0.05 |
| **Male, n (%)** | 715 (41.91) | 337 (43.26) | 0.54 |
| **Black, n (%)** | 978 (57.73) | 307 (39.41) | <0.0001 |
| **White, n (%)** | 464 (27.2) | 341 (43.77) | <0.0001 |
| **Hispanic, n (%)** | 264 (15.47) | 83 (10.65) | 0.0012 |
| **Other, n (%)** | 0 (0.00) | 48 (6.16) | <0.0001 |
| **Married, n (%)** | 772 (45.90) | 353 (49.37) | 0.13 |
| ***Socioeconomic status*** | | | |
| ***Education*** | | | |
| **Less than High School, N (%)** | 286 (16.80) | 67 (9.36) | <0.0001 |
| **High School, N (%)** | 476 (27.97) | 139 (19.41) | <0.0001 |
| **Some College, N (%)** | 815 (47.88) | 425 (59.36) | <0.0001 |
| **College or higher, N (%)** | 125 (7.34) | 85 (11.87) | 0.0005 |
| ***Income*** | | | |
| **<$16,000, N (%)** | 328 (21.05) | 106 (16.21) | 0.0098 |
| **$16,000 - $29,999, N (%)** | 298 (19.13) | 79 (12.08) | <0.0001 |
| **$30,000 - $49,999, N (%)** | 435 (27.92) | 191 (29.20) | 0.57 |
| **>$50,000, N (%)** | 497 (31.90) | 278 (42.51) | <0.0001 |
| **Insurance, yes** | 1251 (73.85) | 558 (80.06) | 0.0014 |
| ***CV Risk*** | | | |
| **History of hypertension, N (%)** | 924 (54.16) | 370 (47.50) | 0.0021 |
| **History of diabetes, N (%)** | 299 (17.53) | 108 (13.86) | 0.02 |
| **History of CVD, N (%)** | 106 (6.21) | 16 (5.05) | 0.52 |

*Note:* SD=Standard Deviation, CVD=Cardiovascular Disease
