# Supplementary material for: Unfavorable perceived neighborhood environment associates with less routine healthcare utilization: Data from the Dallas Heart Study
Source: PLoS One. 2020 Mar 12;15(3):e0230041. doi: 10.1371/journal.pone.0230041 (PMC7067436; doi:10.1371/journal.pone.0230041)
Supplement: S4 Table — Reference group reports having one usual source of care. Model adjusted for age, sex, race/ethnicity, marital status, income, education, neighborhood deprivation index, insurance status, cardiovascular disease, comorbid disease burden, depression and experience of discrimination. (DOCX) [file pone.0230041.s004.docx]

Supplemental Table 4. Odds Ratios of Reporting a Usual Source of Care as related to Neighborhood Deprivation Index. Reference group reports having one usual source of care. Model adjusted for age, sex, race/ethnicity, marital status, income, education, neighborhood deprivation index, insurance status, cardiovascular disease, comorbid disease burden, depression and experience of discrimination.

| Usual Source of Care | Odds Ratio Estimate | Confidence Interval |
| --- | --- | --- |
|  | | |
| Yes, one place | Reference Group | |
| Yes, more than one place | 0.97 | 0.69 – 1.36 |
| None | 0.94 | 0.77 – 1.14 |
