# Supplementary material for: Unfavorable perceived neighborhood environment associates with less routine healthcare utilization: Data from the Dallas Heart Study
Source: PLoS One. 2020 Mar 12;15(3):e0230041. doi: 10.1371/journal.pone.0230041 (PMC7067436; doi:10.1371/journal.pone.0230041)
Supplement: S5 Table — Referent group reports having one usual source of care. Model adjusted for age, sex, race/ethnicity, marital status, income, education, insurance status, cardiovascular disease, comorbid disease burden, depression and experience of discrimination. (DOCX) [file pone.0230041.s005.docx]

Supplemental Table 5. Odds Ratios of Reporting a Usual Source of Care as related to Neighborhood Environment Perception. Referent group reports having one usual source of care. Model adjusted for age, sex, race/ethnicity, marital status, income, education, insurance status, cardiovascular disease, comorbid disease burden, depression and experience of discrimination.

|  | Odds Ratio Estimate | Confidence Interval |
| --- | --- | --- |
|  | | |
| **Total Neighborhood Environment Perception** | | |
| Yes, one place | Reference Group | |
| Yes, more than one place | 1.05 | 0.98 – 1.37 |
| None | 1.16 | 0.79 – 1.41 |
|  | | |
| **Factor 1: Perceived Violence** | | |
| Yes, one place | Reference Group | |
| Yes, more than one place | 0.99 | 0.73 – 1.33 |
| None | 1.03 | 0.88 – 1.20 |
|  | | |
| **Factor 2: Perceived Physical Environment** | | |
| Yes, one place | Reference Group | |
| Yes, more than one place | 1.18 | 0.90 – 1.54 |
| None | **1.22** | **1.04 – 1.43** |
|  | | |
| **Factor 3: Perceived Social Cohesion** | | |
| Yes, one place | Reference Group | |
| Yes, more than one place | 0.90 | 0.68 – 1.20 |
| None | 1.05 | 0.88 – 1.24 |
