# Supplementary material for: Unfavorable perceived neighborhood environment associates with less routine healthcare utilization: Data from the Dallas Heart Study
Source: PLoS One. 2020 Mar 12;15(3):e0230041. doi: 10.1371/journal.pone.0230041 (PMC7067436; doi:10.1371/journal.pone.0230041)
Supplement: S6 Table — Reference group reports most recent routine health check-up within past year (0–12 months). Model adjusted for age, sex, race/ethnicity, marital status, income, education, neighborhood deprivation index, insurance status, cardiovascular disease, comorbid disease burden, depression and experience of discrimination. (DOCX) [file pone.0230041.s006.docx]

Supplemental Table 6. Odds Ratios of Reporting Routine Check-up as related to Neighborhood Deprivation Index. Reference group reports most recent routine health check-up within past year (0 – 12 months). Model adjusted for age, sex, race/ethnicity, marital status, income, education, neighborhood deprivation index, insurance status, cardiovascular disease, comorbid disease burden, depression and experience of discrimination.

| Time Since Last Check-up | Odds Ratio Estimate | Confidence Interval |
| --- | --- | --- |
|  | | |
| 0 – 12 months | Reference Group | |
| 1 – 2 years | 0.79 | 0.63 – 0.98 |
| 2 – 5 years | 1.13 | 0.87 – 1.46 |
| More than 5 years or Never | 0.77 | 0.59 – 1.01 |
