# Supplementary material for: Unfavorable perceived neighborhood environment associates with less routine healthcare utilization: Data from the Dallas Heart Study
Source: PLoS One. 2020 Mar 12;15(3):e0230041. doi: 10.1371/journal.pone.0230041 (PMC7067436; doi:10.1371/journal.pone.0230041)
Supplement: S7 Table — Reference group reports most recent routine health check-up within past year (0–12 months). Model adjusted for age, sex, race/ethnicity, marital status, income, education, insurance status, cardiovascular disease, comorbid disease burden, depression and experience of discrimination. (DOCX) [file pone.0230041.s007.docx]

Supplemental Table 7. Odds Ratios of Reporting Routine Check-up as related to Neighborhood Environment Perception and Subfactors. Reference group reports most recent routine health check-up within past year (0 – 12 months). Model adjusted for age, sex, race/ethnicity, marital status, income, education, insurance status, cardiovascular disease, comorbid disease burden, depression and experience of discrimination.

|  | Odds Ratio Estimate | Confidence Interval |
| --- | --- | --- |
|  | | |
| **Total Neighborhood Environment Perception** | | |
| 0 – 12 months | Reference Group | |
| 1 – 2 years | 1.00 | 0.83 – 1.20 |
| 2 – 5 years | **1.34** | **1.07 – 1.68** |
| More than 5 years or Never | **1.33** | **1.09 – 1.62** |
|  | | |
| **Factor 1: Perceived Violence** | | |
| 0 – 12 months | Reference Group | |
| 1 – 2 years | 1.09 | 0.92 – 1.29 |
| 2 – 5 years | **1.33** | **1.09 – 1.63** |
| More than 5 years or Never | 1.14 | 0.94 – 1.38 |
|  | | |
| **Factor 2: Perceived Physical Environment** | | |
| 0 – 12 months | Reference Group | |
| 1 – 2 years | 0.97 | 0.81 – 1.16 |
| 2 – 5 years | **1.30** | **1.04 – 1.62** |
| More than 5 years or Never | **1.36** | **1.13 – 1.65** |
|  | | |
| **Factor 3: Perceived Social Cohesion** | | |
| 0 – 12 months | Reference Group | |
| 1 – 2 years | 0.94 | 0.80 – 1.12 |
| 2 – 5 years | 1.11 | 0.88 – 1.41 |
| More than 5 years or Never | 1.17 | 0.95 – 1.44 |
