# Supplementary material for: Unfavorable perceived neighborhood environment associates with less routine healthcare utilization: Data from the Dallas Heart Study
Source: PLoS One. 2020 Mar 12;15(3):e0230041. doi: 10.1371/journal.pone.0230041 (PMC7067436; doi:10.1371/journal.pone.0230041)
Supplement: S8 Table — (DOCX) [file pone.0230041.s008.docx]

**Supplemental Table 8. Factor Analysis for Creation of Neighborhood-Related Factors in the Dallas Heart Study**

| **Component** | **Question pertaining to:** | **Mean (SD)** | **Factor 1** | **Factor 2** | **Factor 3** |
| --- | --- | --- | --- | --- | --- |
| Violence (Factor 1) | Gang violence | 1.53 (1.04) | 0.75 | 0.17 | 0.08 |
|  | Fights with weapons | 2 (1.35) | 0.74 | 0.25 | 0.15 |
|  | Violent arguments | 2.02 (1.34) | 0.67 | 0.28 | 0.19 |
|  | Sexual assault | 1.46 (0.92) | 0.67 | 0.12 | 0.06 |
|  | Robbery | 2.16 (1.35) | 0.62 | 0.15 | 0.20 |
| Physical Environment (Factor 2) | Trash and litter | 2.12 (1.45) | 0.22 | 0.74 | 0.12 |
|  | Lack of recreation areas | 2.08 (1.45) | 0.10 | 0.66 | 0.06 |
|  | Food shops | 1.88 (1.36) | 0.14 | 0.64 | 0.01 |
|  | Sidewalks | 2.02 (1.42) | 0.11 | 0.63 | 0.05 |
|  | Excessive noise | 2.23 (1.43) | 0.25 | 0.56 | 0.21 |
|  | Heavy traffic | 2.5 (1.52) | 0.21 | 0.54 | 0.19 |
| Social Cohesion (Factor 3) | Willingness to help neighbors | 2.4 (1.26) | 0.17 | 0.09 | 0.05 |
|  | Close knit neighborhood | 2.75 (1.39) | 0.07 | 0.06 | 0.21 |
|  | People trusted | 2.68 (1.33) | 0.27 | 0.18 | 0.19 |
|  | | Eigenvalue | 10.52 | 2.39 | 1.84 |
|  |  | Common variance explained by each component | 0.76 | 0.17 | 0.13 |
|  |  | Cronbach’s alpha coefficient | 0.84 | 0.82 | 0.76 |
